# Impact of allergic rhinitis and specific subcutaneous immunotherapy on peripheral blood basophils of patients sensitized to *Dermatophagoides pteronyssinus*

**DOI:** 10.1186/1710-1492-9-40

**Published:** 2013-10-11

**Authors:** Ana Lopes, Patrícia Azenha, Cristina Teodósio, Maria Inácio, Isabel Silva, Graça Loureiro, António Martinho, António S Luís, Hélder Trindade, Celso Pereira, Artur Paiva

**Affiliations:** 1 Blood and Transplantation Center of Coimbra, Portuguese Institute of Blood and Transplantation, Edifício São Jerónimo, 4 Piso, Praceta Mota Pinto, 3001-301, Coimbra, Portugal; 2 College of Health Technology of Coimbra, São Martinho do Bispo, 3046-854, Coimbra, Portugal; 3 General Cytometry Service, Cancer Research Center (IBMCC-CSIC/USAL and IBSAL) and Department of Medicine, University of Salamanca, Salamanca, Spain; 4 Immunoallergology Department, University Hospital of Coimbra, 3000-075, Coimbra, Portugal

**Keywords:** Allergic rhinitis, *Dermatophagoides pteronyssinus*, Basophils, *Histamine N-methyltransferase*, Tryptase, Specific subcutaneous immunotherapy

## Abstract

**Background:**

Basophils are important effectors cells in allergic rhinitis (AR) since they are involved in immunoglobulin (Ig) E – mediated inflammation and in the release of pro-inflammatory mediators. Specific subcutaneous immunotherapy (SCIT) provides clear immunologic modulation in some immune cells, however its systemic effects on basophils are not well known.

**Methods:**

Peripheral blood (PB) samples from 43 patients with allergic rhinitis mono-sensitized to *Dermatophagoides pteronyssinus* (Dpt) [33 of them under SCIT with allergoid Dpt extract, in maintenance dose (SCIT), with evaluation just before SCIT injection (SCIT-T0) and 4 hours later (SCIT-T4) and the other 10 Dpt allergic patients never having, in the past, undergone specific immunotherapy treatment (NSIT)], and 15 healthy age- and gender-matched controls (HG), were analyzed. For each sample, the total (t-IgE) and specific IgE (s-IgE) was performed, as well as, the relative frequency and absolute number of PB basophils and receptor-bound IgE and IgG expression were evaluated by flow cytometry and the *Histamine N-methyltransferase* (*HNMT*) and tryptase α/β1 (*TPSAB1*) gene expression was assessed by real-time PCR.

**Results:**

Higher levels of receptor-bound IgE were observed in SCIT patients, which are correlated with the levels of serum t-IgE and s-IgE, whereas no significant differences were observed for receptor-bound IgG. Regarding *HNMT* mRNA expression, significantly lower expression levels were detected in AR patients compared to HG, independently of type of therapy. Moreover a negative correlation was found between *HNMT* gene expression and time under SCIT. Conversely, tryptase gene expression was significantly up-regulated in NSIT when compared to HG; however in SCIT patients, tryptase gene expression was significantly decreased than in NSIT patients. No differences were found for any parameter between SCIT-T0 and SCIT-T4 with exception of a transient increased expression of tryptase in SCIT-T4.

**Conclusion:**

PB basophils from patients with AR show altered functional features, which seems to be influenced by SCIT, suggesting that these cells could be useful to clarify the SCIT triggered mechanisms at a systemic level.

## Introduction

Rhinitis is a heterogeneous group of nasal disorders characterized by one or more symptoms, including, sneezing, itching, rhinorrhea, and/or nasal congestion, which ultimately have a negative impact on the patient’s quality of life. Rhinitis can be caused by nonallergic factors like infection, hormones or others [1,2], although approximately 50% of all cases are allergy-related. This later variant of the disease - allergic rhinitis (AR) – is the most common allergic condition affecting 10% to 40% of the world population, depending on the geographical setting [3].

Several factors may specifically contribute to the pathophysiology of AR, including the local synthesis of immunoglobulin (Ig) E in nasal mucosa, the up-regulation of Th2 cytokines or the alteration in the number of regulatory T (Treg) cells [4]. However, as an allergic process, most symptoms arise as a consequence of local inflammation induced by an IgE-mediated immune response to specific allergens such as pollens, molds, animal dander and dust mites [5], ultimately leading to the local accumulation of an inflammatory cell infiltrate composed of different cells including T cells, eosinophils, mast cells and basophils [6,7]. As a result of the cross-linking of IgE on basophils and mast cells by allergens, several mediators (e.g. leukotrienes, prostaglandins, tryptase and histamine) are released contributing to the development of the inflammatory response and the symptoms [8].

Basophils along with eosinophils and mast cells, are the main effector cells in this allergic inflammation process, but unlike the later cells that reside in the tissue, basophils circulate in the peripheral blood and selectively migrate to sites of inflammation [9,10]. Additionally, their active participation in the pathogenesis of allergic inflammation has been widely supported in the literature through the release of preformed and newly generated mediators like leukotriene C_4_, major basic protein, IL-4, IL-13, tryptase and histamine [11-15].

Among these molecules, histamine is the primary mediator involved in the development of symptoms of AR in both early and late-phase reactions, being also able to activate immunocompetent cells, including T lymphocytes influencing the Th1/Th2 balance towards a Th2 polarization [16]. The regulation of the production and activity of this mediator is performed by its inactivation and/or transport into cells to remove an excess amount of histamine in extracellular space. The major routes of histamine inactivation are catalyzed by two main catabolic pathways: methylation by oxidative deamination by diamine oxidase (DAO) and histamine N-methyltransferase (HNMT), which has been suggested to play a critical role in the degradation of histamine in the airway [17,18].

Specific subcutaneous immunotherapy (SCIT) has been widely reported to show clinical effectiveness in the treatment of allergic rhinitis and asthma [19], providing a long-term remission of allergic symptoms and reducing the development of new sensitization to other allergens [20,21]. The mechanisms by which clinical responses are achieved have been extensively described and include a decrease in basophils and mast cell activation within the affected tissues, induction of T cells anergy, generation of Th1 and Treg cells, decrease of allergen-specific IgE antibody levels, associated with the production of allergen-specific IgG_1_ and IgG_4_ antibodies (IgG “blocking activity”) and induction of the production of non-inflammatory IgA antibodies by B cells [20,22-24].

Interestingly, despite the influence of AR on effector cells, like basophils, and the impact of SCIT therapy within the nasal mucosa, are relatively well known, the potential systemic effect of AR and SCIT on PB basophils remains largely unknown.

In this context, we mainly aimed to evaluate the effects of AR and SCIT on PB basophils. For this propose we analyzed the frequency of PB basophils, expression of receptor-bound IgE and IgG on the membrane and the mRNA expression of basophil mediator-related genes (*histamine N-methyltransferase* and tryptase) in patients with monosensitization to *Dermatophagoides pteronyssinus* (Dpt) submitted to SCIT (before and 4 hours later the allergen injection) and compared them to AR patients who were never submitted to SCIT and to healthy individuals. Moreover we looked for a possible correlation between those results with total (t-IgE) and specific IgE (s-IgE) in the serum and with time under SCIT.

## Materials and methods

### Patients

Overall, 43 AR patients were included in this study, all of them with at least 2 years of perennial symptoms, wheal 3 mm larger than the negative control related to histamine response and the values of serum specific IgE at least class 2 (CAP). 33 patients with AR mono-sensitized to mites under active maintenance SCIT treatment with allergoid Dpt extract Depigoid (Leti, Madrid, Spain) with monthly maintenance dose of 0.5 ml volume (Der p 1 = 7.2 μg/ml) (SCIT) (age: 30 ± 11 years; sex: 19 males and 14 females) (Table 1). The diagnosis was established based on clinical symptoms, positive skin prick tests, serum specific IgE assay, and positive nasal challenge test.

**Table 1:** Clinical and laboratorial characteristics of the individuals included in the study

	**HG**	**NSIT**	**SCIT**
	**(n = 15)**	**(n = 10)**	**(n = 33)**
Age (years)			
Mean ± SD	29 ± 10	26 ± 7	30 ± 11
Range	(18–38)	(19–38)	(17–61)
Gender			
Male	53% (n = 8)	30% (n = 3)	58% (n = 19)
Female	47% (n = 7)	70% (n = 7)	42% (n = 14)
Clinical parameters			
Time from disease onset (years)	NA	10 ± 7	12 ± 8
Time of SCIT (months)	NA	NA	28 ± 13
Presence of Asthma	NA	40% (4/10)	43% (13/30)
Laboratorial parameters			
Serum total IgE (t-IgE) (kU/L)	NA	295 ± 231	514 ± 787
Serum specific IgE (s-IgE) (kU_A_/L)	NA	48 ± 27	45 ± 35

*NA,* Not applies. The results were given by mean ± standard deviation.

Exclusion criteria included the following: pregnancy, pediatric group, mental diseases or other comorbidities, chronic medication and/or active infection and inflammation. At the moment of the treatment implementation a diagnosis of persistent moderate/severe rhinitis, ARIA Classification [25], and the presence of concomitant mild persistent controlled asthma, GINA Classification [26] was not an exclusion criteria. When included in the study, all patients presented clinical efficacy to the treatment, as defined by reduction of clinical score and medication consumption such as inhaled steroids and relief medication (oral anti-histamines). In all of them the induction phase was achieved after 6 doses of weekly injection, followed by 1 injection every 4 weeks as a maintenance dose for a minimum of 12 months. For this particular group of patients an evaluation before SCIT injection (SCIT-T0) and 4 hours later (SCIT-T4) was performed, according the time of the beginning of the late phase of allergen inflammatory response [27].

Additionally, a control group (NSIT) of 10 respiratory allergic patients monosensitized to mites who had not submitted, in the past, to specific immunotherapy, and that displayed the same clinical profile and the exclusion criteria as the SCIT group (age: 26 ± 7 years; sex: 3 males/7 females) was studied (Table 1). Treatment in this group of patients was implemented according to the recommended guidelines (oral systemic anti-histamines, nasal and bronchial corticosteroid therapy and bronchodilators for patients with asthma). Of note, all the analyses were performed during a period of clinical stabilization in both AR groups.

### Healthy individuals group

Another control group was selected composed by 15 healthy individuals (HG) (age: 29 ± 10 years; sex: 8 males/7 females). These participants were required to complete a brief questionnaire regarding previous or current medical conditions. Inclusion criteria for this group included absence of autoimmune and allergic diseases, as well as, of an active infection. Additionally, only individuals who were not undergoing treatment with immunomodulatory drugs for any known conditions were included in this control group.

### Samples

PB samples from each individual were collected into K3-EDTA, serum tubes (Becton Dickinson (BD) Bioscience, San Jose, CA, USA) and PAXgene Blood RNA tubes (PreAnalytiX GmbH, Switzerland).

#### ***Ethical standards***

The study protocol was approved by the Ethical Committee from Coimbra 185 University Hospital. All participants gave and signed an informed consent form, and the principles of Helsinki Declaration were respected.

#### ***Determination of total and specific IgE***

Quantitative determination of serum t-IgE was performed using nephelometry method. Total IgE concentration (kU/L) was determined comparing the results with the calibrators provided.

Serum s-IgE to *Dermatophagoides pteronyssinus* was determined using the ImmunoCAP fluorescence enzyme immunoassay technique (Phadia, Uppsala, Sweden). To evaluate the test results, the response from the patients samples was converted to concentrations using a calibration curve. The range of measurements was 0.35 to 100 KU_A_/L.

#### ***Multiparameter flow-cytometry immunophenotypic studies of PB basophils***

Identification and characterization of PB basophils was performed using a anti-IgG conjugated with flourescein isothiocyanate (clone: G18-145, BD Bioscience), anti- IgE conjugated with phycoeritrin (clone: BE5, EXBIO Praha, Vestec, Czech Republic), anti-HLA-DR peridinin chlorophyll protein cyanine 5.5 (clone: G46-6, BD Bioscience), anti-CD123 allophycocyanin (clone: AC145, Miltenyi Biotec; Bergisch, Gladbach, Germany) and anti-CD45 krome orange (clone: J.33, Beckman Coulter, Brea, CA, USA) combination of monoclonal antibodies (mAb).

For sample processing, two washing steps using phosphate-buffered saline (PBS) (Gibco BRL-life Technologies, Vienna, Austria) were performed before staining, in order to remove the free immunoglobulins present in the plasma samples. For sample staining, a direct immunofluorescence technique was used. Briefly, mAb were added to 200 μL of PB and were incubated for 15 min at room temperature in darkness. After this incubation period, a lyse-and-then-wash protocol was followed: incubation with 2 mL of FACS Lysing Solution (BD Bioscience) diluted 1:10 (vol/vol) for 10 min followed by a washing step with 2 mL of PBS (Gibco BRL-life Technologies). Cells were ressuspended in 0.5 mL of PBS (Gibco BRL-life Technologies) before acquisition in flow cytometer.

#### ***Flow cytometry data acquisition and analysis***

Data acquisition was performed in a FACSCanto II flow cytometer (BD Bioscience) using the FACSDiva software (BD Bioscience).

Basophils were identified according to their high expression of CD123 in absence of HLA-DR and by their moderate CD45 expression, dimmer than lymphocytes [28]. The relative amount of receptor-bound IgE and IgG was determined based on their mean fluorescence intensity (MFI).

Absolute numbers were calculated using a dual platform methodology (flow cytometry and hematological cell analyser). For data analysis the Infinicyt™ software, V.1.5 (Cytognos SL, Salamanca, Spain) was used.

#### ***Gene expression analysis***

For the analysis of *histamine N-methyltransferase* (*HNMT*) and tryptase α/β1 (*TPSAB1*) gene expression, whole peripheral blood was collected in a PAXgene Blood RNA Tube (PreAnalytiX) and total automated RNA purification was performed in QIAcube (Qiagen, Hilden, Germany).

RNA integrity and quantification were performed using a 6000 Nano Chip^®^ Kit in an Agilent 2100 bioanalyzer (Agilent, Walbronn, Germany). For quantitative reverse-transcription polymerase chain reaction (RT-qPCR), one microgram of RNA was reverse transcribed with iScript™ Reverse Transcription Supermix (Bio-Rad, Hercules, CA, USA) according to the manufacturer’s instructions.

Relative quantification of gene expression was performed by real-time PCR using the LightCycler 480 II thermocycler (Roche, Basel, Switzerland). Normalization for gene expression quantification was performed with a geNorm Housekeeping Gene Selection Human Kit (Primer Design, Southampton, UK) and geNorm software (Center for Medical Genetics, Ghent University Hospital, Ghent, Belgium), in order to select optimal housekeeping genes for this study [29].

Real-time PCR reactions performed using specific QuantiTect Primer Assays (Qiagen) with optimized primers for *HNMT* (QT00009282) and *TPSAB1* (QT01670298) and endogenous controls as *beta-actin* (QT01680476) and *glyceraldehyde 3-phosphate dehydrogenase* (QT01192646) with QuantiTect SYBR Green PCR Kit Gene expression (Qiagen), according to the manufacturer’s instructions. Reactions were performed with the following thermal cycling profile: 10 min at 95°C, plus 50 cycles of 10 s at 95°C, 20 s at 55°C, and 30 s at 72°C. Quantitative real-time PCR results were analyzed with LightCycler 480 software (Roche) and quantification was performed using the qBasePlus software package (Biogazelle, Zulte, Belgium).

#### ***Statistical analyses***

Statistical evaluations of data were performed using the non-parametric Mann–Whitney U test for independent variables. Wilcoxon signed-rank test was used to compare related groups and a Spearman’s rank correlation was applied to detect the association between different parameters. Results were expressed as median with interquartile range. All statistical analyses were performed using Statistical Package for Social Sciences IBM SPSS 20 (IBM, Armonk, NY. USA) and Graphpad Prism version 5 (GraphPad Software, San Diego, CA, USA). Differences were considered to be statistically significant when the *p* value was less than 0.05.

## Results

### Relative and absolute quantification of PB basophils

The relative frequency and absolute number of PB basophils were similar among the four studied groups (Table 2). Of note, no significant association was found, both for the frequency and absolute number of PB basophils with the time under SCIT (data not show).

**Table 2:** Relative frequency and absolute cell number of circulating peripheral blood basophils in the studied groups

	**HG**	**NSIT**	**SCIT-T0**	**SCIT-T4**
Relative frequency (%)	0.44	0.40	0.49	0.39
	(0.27 – 0.74)	(0.25 – 0.67)	(0.17 – 0.85)	(0.17 – 1.1)
Absolute number (cell/μL)	34	36	41	38
	(21 – 70)	(17 – 56)	(11 – 70)	(15 – 97)

Differences were considered statistically significant when *p <* 0.05. The Mann–Whitney U test used to compare between HG, NSIT, SCIT [evaluation before Dpt injection (T0) and 4 hours late (T4)]. Wilcoxon signed-rank test was used to compare SCIT-T0 *versus* the SCIT-T4 group. The results were given by median with interquartile range.

### Serum total and specific IgE levels

Slightly lower total serum IgE (t-IgE) levels were found in NSIT patients when compared with the SCIT patients, although not reaching statistical significance. Conversely, no differences were observed between the two AR groups regarding serum specific IgE (s-IgE) levels (Table 1).

### Expression of receptor-bound IgE and IgG on the membrane of PB basophils

**Figure 1:**
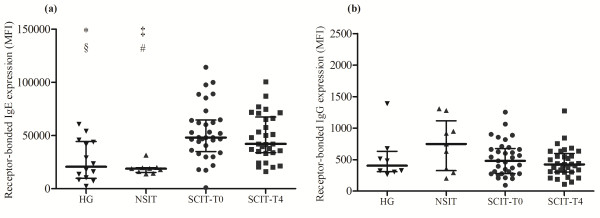
**Receptor-bound IgE (a) and IgG (b) on PB basophils.** Statistical significant differences were considered when p < 0.05. Mann–Whitney *U* test was used to compare between HG, NSIT, SCIT [evaluation before Dpt injection (T0) and 4 hours later the SCIT infusion (T4)]. Wilcoxon signed-rank test was used to compare SCIT-T0 *versus* the SCIT-T4 group. The results were given by median with interquartile range. * HG *versus*  SCIT-T0, § HG *versus* SCIT-T4 , # NSIT *versus* SCIT-T0, ‡ NSIT *versus* SCIT-T4.

**Figure 2:**
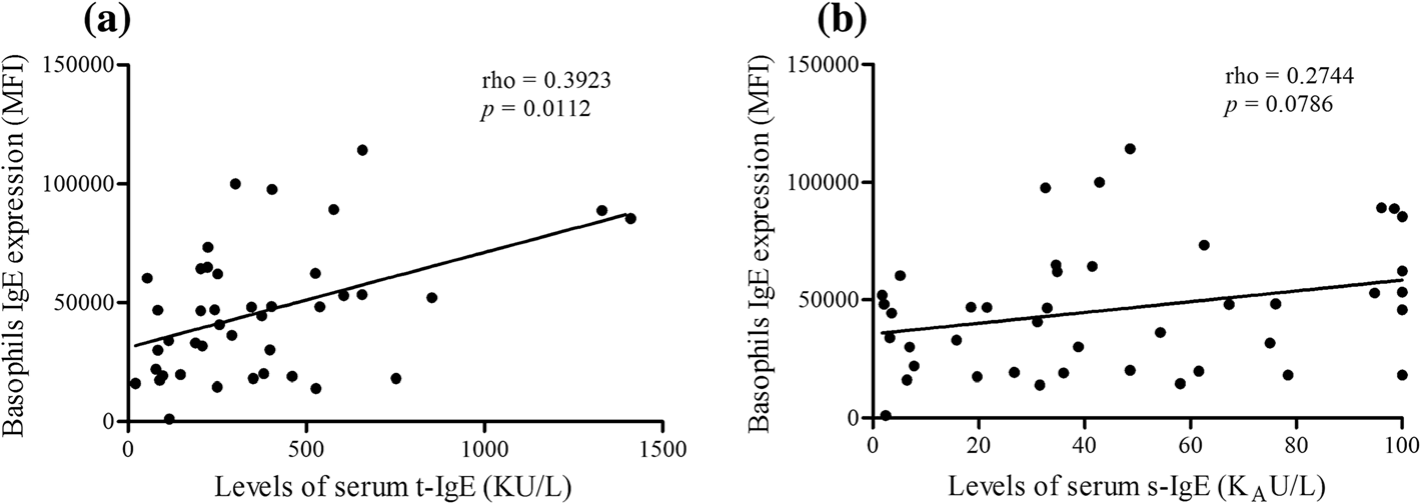
**Corelation between receptor-bound IgE and serum t-IgE (a) and s-IgE (b). (a)** Positive correlation (rho=0.3923) with statistical significance (p=0.0112); **(b)** Absence of correlation (rho=0.2744; p=0.0786). Statistical significant differences were considered when p < 0.05. The correlations were assessed by the Spearman’s rank correlation.

Receptor-bound IgE expression was significantly increased, and more heterogeneous, in the SCIT patients, compared with both HG and NSIT (Figure 1a). In line with this observation, a significant association was found between serum t-IgE and s-IgE levels and receptor-bound IgE expression, thus patients with higher serum t-IgE levels and/or s-IgE levels also showed higher IgE receptor-bound levels on basophil membrane (Figure 2).

Regarding receptor-bound IgG, no significant differences were detected among the studied groups, with AR patients showing expression levels similar to healthy individuals. Nevertheless, NSIT patients showed a very heterogeneous distribution, with a slight tendency for a higher expression compared to both HG and SCIT (Figure 1b). No differences were found for both receptor-bound IgE and IgG, comparing SCIT-T0 and SCIT-T4 patients.

Furthermore, a significantly decreased ratio of receptor-bound IgG and IgE was observed in SCIT patients compared to NSIT patients, independently of time after SCIT injection (Table 3).

**Table 3:** ***Ratio *of receptor-bound IgG and IgE on the membrane of PB basophils**

	**HG**	**NSIT**	**SCIT-T0**	**SCIT-T4**
*Ratio* IgG/IgE	2.23	3.50	0.96*	0.98^#^
	(0.56 – 13.57)	(1.01 – 8.98)	(0.35 – 2.28)	(0.17 – 3.81)

Differences were considered statistically significant when *p <* 0.05. The Mann–Whitney U test used to compare between HG, NSIT, SCIT [evaluation before Dpt injection (T0) and 4 hours late (T4)]. Wilcoxon signed-rank test was used to compare SCIT T0 *versus* the SCIT T4 group. The results were given by median with interquartile range.

*SCIT-T0 *versus* NSIT. ^#^SCIT-T4 *versus* NSIT.

### Histamine N-methyltransferase (HNMT) and tryptase α/β 1 (TPSAB1) gene expression in whole peripheral blood

**Figure 3:**
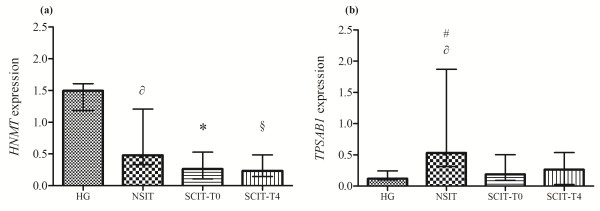
***HNMT *(a) and *TPSAB1 *(b) expression in whole PB.** Statistical significant differences were considered when p < 0.05. Mann–Whitney *U* test was used to compare between HG, NSIT, SCIT [evaluation before Dpt injection (T0) and after 4 hours (T4)]. Wilcoxon signed-rank test was used to compare SCIT-T0 *versus* the SCIT-T4 group. The results were given by median with interquartile range. * HG *versus* SCIT-T0, § HG *versus*  SCIT-T4 , ∂ HG *versus* NSIT, # NSIT *versus* SCIT-T0.

Overall, AR patients showed significantly lower *HNMT* expression levels when compared with healthy controls, which was more evident in SCIT patients. 4 hours after SCIT injection the expression of *HNMT* remains unchanged (Figure 3a).

Concerning tryptase gene expression significant differences were found between groups. Its expression was increased in NSIT compared to HG. On the other hand, SCIT group showed a significant decreased of tryptase gene expression than NSIT group (Figure 3b). Despite not reaching statistical significance, in the SCIT group, tryptase gene expression showed a tendency to increase 4 hours after SCIT injection.

### Effect of treatment on serum total and specific IgE, receptor-bound IgE and IgG and HNMT and TPSAB1 gene expression levels according time

**Figure 4:**
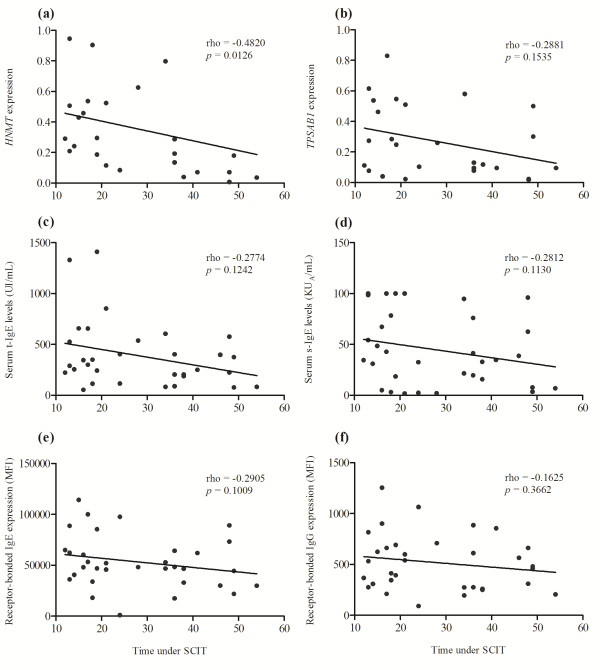
**Correlation between analytical parameters and time under SCIT. (a)** Negative correlation between *HNMT* expression and time under SCIT (rho=-0.4820; p=0.0126). Absence of correlation between time under SCIT and *TPSAB1* expression **(b)**, serum t-IgE levels **(c)**, serum s-IgE levels **(d)**, receptor-bounded IgE **(e)** and receptor-bounded IgG **(f)**. Statistical significant differences were considered when p < 0.05. Correlations were assessed by the Spearman’s rank correlation. The evaluation was performed in SCIT patients before SCIT injection.

*HNMT* gene expression was negatively correlated (p = 0.0126) with the time under SCIT. There was a similar also effect on *TPSAB1* gene expression, although the difference did not reach statistical significance (Figure 4a, b).

Similarly, serum t-IgE and s-IgE levels had a trend to decrease over time with SCIT, however it did not reach statistical significance (Figure 4c, d). Likewise, little negative correlation and an absence of correlation were found for receptor-bound IgE and IgG expression over time with SCIT, respectively (Figure 4e, f).

## Discussion

Specific immunotherapy currently represents the only treatment that modifies the natural history of allergic rhinitis [27] with proven clinical effectiveness [30]. However, despite the effects of this treatment being relatively well described, including important immunologic changes, like a modulation of the T-cell profile, with induction of allergen-specific Treg cells, that inhibit a proliferative and cytokine response to the allergen [31,32] or a local decrease in mast cell and basophil activity [33,34]. The systemic impact of SCIT in AR patients mono-sensitized to Dpt, as well as, the repercussion of long-term treatment on the effector cells of the allergic inflammation, are widespread under research. To address this issue, in this study we analyzed the frequency and absolute number, the levels of receptor-bound IgE and IgG and the expression of mediator-related genes of circulating PB basophils from AR patients undergoing SCIT and compared them with both healthy individuals and AR patients who had never been treated with SCIT.

Overall our results suggest that, contrary to what has been described for other allergic disorders, like hymenoptera allergy [35], AR patients did not show increased PB basophil numbers. In fact, no significant differences were found for both relative and absolute basophil frequency between the different groups included in the study. These findings are probably reflecting a distinct route of exposure of the allergen in allergic rhinitis and are in line with previous studies which reported an increased frequency of basophils locally, within the airways, in allergic rhinitis [36,37]. Overall, these results further suggest that, in allergic rhinitis, the high-turnover state of the basophil progenitors, resulting the short span of these cells, could balance their levels in PB circulation [38].

Regarding the amount of receptor-bound IgE on the basophils surface, increased and more heterogeneous expression levels were found in the SCIT group compared to both NSIT and HG, which showed a significant association with serum IgE levels. These observations are in agreement with previous studies by Kawakami *et al*. which reported a relationship between basophil expression of high-affinity IgE receptor (FcϵRI) and serum IgE levels, as IgE binding to its high affinity receptor leads to increased expression of FcϵRI [23,39-41]. Altogether, these results seem to suggest that, since patients with higher levels of serum t-IgE and s-IgE exhibited increased FcϵRI-bound IgE expression, the increased values for receptor-bound IgE observed in these patients are also reflecting an increased expression of FcϵRI on the basophils membrane. This is in line with previous studies, which described increased FcϵRI expression in allergic patients [42,43].

Interestingly, both receptor-bound IgE expression and serum IgE levels were more heterogeneous in SCIT patients, compared to both HG and NSIT. Since it is known that allergen-specific immunotherapy leads to an initial rise, followed by a slow decline, in the level of serum IgE in allergic patients, during SIT, this high variability could reflect differences in the time that patients were undergoing SCIT. In fact, when we analysed the SCIT effect in the levels of serum t-IgE and receptor-bound IgE expression, we observed lower levels, for both parameters, in patients under more time of SCIT [44].

Interestingly, despite it is widely known that allergen-specific immunotherapy leads to an increase in serum specific IgG_4_, similar levels of expression were found for all the groups included in the study regarding the amount of receptor-bound IgG on the membrane of PB basophils. This could probably due to the fact that the anti-IgG antibody used to detect bound-IgG also detects other IgG isotypes. Further studies would be necessary, using antibodies for allergen specific IgG and its isotypes, not only in basophils, but also on other PB cells which express IgG receptors, to determine whether the increased of IgG_4_ serum levels are associated with a change in the membrane-bound IgG_4_ or other isotypes expression or, as it has been previously suggested, if the IgG_4_ produced during specific immunotherapy does not bind the effector cells, but protects the patients by binding the allergen, and therefore preventing its cross-linking to allergen specific IgE attached to effector cells or by forming IgG_4_-allergen-IgE complexes, ultimately inhibiting IgE triggering [45].

Remarkably, more than a variation in the isotype (IgE *vs.* IgG) or the amount of receptor-bound immunoglobulin on PB basophils, significant differences were observed amongst the different study groups regarding the expression of allergy mediator-related genes. In our study, AR patients showed significantly lower *HNMT* mRNA expression in whole PB, which is in line with previous studies which reported a decreased expression of this enzyme in the nasal mucosa [46]. Since no association was found between the frequency of PB basophils and therapy, this seems to indicate, that the beneficial effects of SCIT on histamine-related symptoms could be due not to a variation in the number of circulating basophils but as a result of histamine lower production. In line with this, previous studies have also reported increased expression of histidine decarboxylase in AR patients nasal mucosa, a key enzyme for the production of histamine [46], and a decreased histamine release as a result of specific immunotherapy to hymenoptera venom [47]. A relevant finding in our study was a negative correlation between time under SCIT and *HNMT* gene expression and since the immunotherapy could modify the histamine metabolism, the decrease of *HNMT* gene expression seems to be an effect of SCIT.

Additionally, histamine metabolism may not be affected directly by allergen-specific immunotherapy, as reported by Novak *et* al*.*, in a model of specific venom immunotherapy, whereas an increase in the expression of histamine receptor type 2 (H2R) in basophils was observed [48,49]. Furthermore, this imbalance in the expression of H2R seems to have a suppressive effect in basophil activity, associated with lower histamine production [49,50].

Concerning tryptase, a more apparent modulation of the gene expression was verified, since a significant up-regulation in NSIT patients occurs compared both to healthy group and SCIT patients. This suggests that SCIT therapy, indeed, leads to a normalization of the production of this mediator, which is in accordance with previous studies by Klimer *et al.* who reported that the higher levels of tryptase in allergic rhinitis patients appear to be significantly inhibited by specific immunotherapy [34]. However, upon administration of SCIT, a slight increase in the expression of this mediator is observed, at least 4 hours later. This observation is in line with the increased expression of activation-related molecules in PB basophils observed immediately upon venom immunotherapy [51], and further supports the generalized recommendation for a mandatory observation period following each injection, in order to monitor the patient for the development of symptoms of type 1 hypersensitivity reaction [52]. Despite this increase after 4 hours, in our patients, SCIT therapy has led to a decrease in the tryptase mRNA expression. Noteworthy, the expression of this mast cell and basophil specific mediator, could be the result of a decrease in the release of basophil mediators [35], this decrease probably reflects the generation of allergen-specific Treg cells that are able to produce anti-inflammatory cytokines like IL-10 [33], known to reduce basophil responsiveness to IgE-mediated stimuli, through the down-regulation of the expression of key IgE signalling molecules [53].

## Conclusion

In summary, we showed that in AR patients under SCIT, peripheral blood basophils are functionally altered, with an increase of the receptor-bound IgE expression which is positively correlated with serum t-IgE and a decrease in *HNMT* and tryptase gene expression, when compared to AR patients never underwent specific immunotherapy. Moreover a significant negative correlation between *HNMT* gene expression and time under therapy was observed.

Overall, these findings seem to contribute to a better definition of the SCIT triggered mechanisms in circulating basophils. However further studies, in a larger cohort of patients, would be necessary to determine the exact role that SCIT therapy could have on these parameters.

## Abbreviations

AR: Allergic rhinitis; Ig: Immunoglobulin; SCIT: Specific subcutaneous immunotherapy; PB: Peripheral blood; Dpt: *Dermatophagoides pteronyssinus*; SCIT-T0: Evaluation just before SCIT injection; SCIT-T4: Evaluation 4 hours after SCIT injection; NSIT: Allergic patients never having undergone specific immunotherapy treatment; HG: Healthy individuals; t-IgE: Serum total IgE; s-IgE: Serum specific IgE; HNMT: Histamine N-methyltransferase; TPSAB1: Tryptase α/β1; Th: T helper cells; Treg: Regulatory T cells; IL: Interleukin; DAO: Diamine oxidase; ARIA Classification: Allergic Rhinitis and its Impact on Asthma; GINA Classification: Global Initiative for Asthma classification; K3-EDTA: Tripotassium ethylene diamine tetraacetic acid; mAb: Monoclonal antibodies; PBS: Phosphate-buffered saline; FACS: Fluorescence-activated cell sorter; MFI: Mean fluorescence intensity; FcϵRI: High-affinity IgE receptor; H2R: Histamine receptor type 2; NA: Not applies.

## Competing interests

The authors declare that they have no competing interests.

## Authors’ contributions

AL carried the analysis and interpretation of data, the statistical analysis and drafted the manuscript. PA and AM participated in the molecular studies. MI and IS performed flow cytometry assays. CT has been involved in manuscript revising. GL, BT and ASL contributed with provision of study material or patients. HT, CP and AP contributed to conception and designed the study protocol and given final approval of the version to be published. All authors read and approved the final manuscript.
